# A Review of Measurement and Characterization of Film Layers of Perovskite Solar Cells by Spectroscopic Ellipsometry

**DOI:** 10.3390/nano15040282

**Published:** 2025-02-13

**Authors:** Liyuan Ma, Xipeng Xu, Changcai Cui, Tukun Li, Shan Lou, Paul J. Scott, Xiangqian Jiang, Wenhan Zeng

**Affiliations:** 1 School of Manufacturing Engineering, Huaqiao University, Xiamen 361021, China; l.ma@hud.ac.uk (L.M.); xpxu@hqu.edu.cn (X.X.); 2 EPSRC Future Advanced Metrology Hub, University of Huddersfield, Huddersfield HD1 3DH, UK; t.li@hud.ac.uk (T.L.); s.lou@hud.ac.uk (S.L.); p.j.scott@hud.ac.uk (P.J.S.); x.jiang@hud.ac.uk (X.J.); z.wenhan@hud.ac.uk (W.Z.); 3 School of Metrology Measurement and Instrument, China Jiliang University, Hangzhou 310018, China

**Keywords:** perovskite solar cells, spectroscopic ellipsometry, film quality, photoelectric properties, geometrical properties

## Abstract

This article aims to complete a review of current literature describing the measurement and characterization of photoelectric and geometric properties of perovskite solar cell (PSC) film layer materials using the spectroscopic ellipsometry (SE) measurement technique. Firstly, the influence of film quality on the performance of PSCs is combed and analyzed. Secondly, SE measurement technology is systematically introduced, including the measurement principle and data analysis. Thirdly, a detailed summary is provided regarding the characterization of the geometric and optoelectronic properties of the substrate, electron transport layer (ETL), perovskite layer, hole transport layer (HTL), and metal electrode layer using SE. The oscillator models commonly used in fitting film layer materials in PSCs are comprehensively summarized. Fourthly, the application of SE combined with various measurement techniques to assess the properties of film layer materials in PSCs is presented. Finally, the noteworthy direction of SE measurement technology in the development of PSCs is discussed. The review serves as a valuable reference for further enhancing the application of SE in PSCs, ultimately contributing to the commercialization of PSCs.

## 1. Introduction

Hybrid perovskite materials have been widely applied as absorption layers in perovskite solar cells (PSCs) due to their excellent properties, such as an adjustable bandgap (about 1.48–2.3 eV) [1,2,3], long and relatively balanced carrier diffusion length [4], and wide spectral absorption range (about 300–800 nm) [5]. Hence, they are regarded as an ideal photovoltaic material. In recent years, the efficiency of PSCs has continuously increased. In 2024, the highest efficiency achieved by a silicon–perovskite tandem solar cell was 34.6%. This record was set by LONGi Green Energy and certified by the European Solar Test Installation (ESTI) [6]. PSCs have garnered significant attention in renewable energy due to their simple preparation process, low cost, and high photoelectric conversion efficiency [7,8].

As a multilayer film stack structure, PSCs’ excellent photoelectric conversion performance depends on film materials and the deposition quality of each film layer. The basic structure of typical PSCs includes the transparent substrate, transparent conductive oxide (TCO), electron transport layer (ETL), perovskite layer, hole transport layer (HTL), and electrode material [9]. To obtain higher photoelectric conversion efficiency, PSCs usually add some additional interface layers [10,11]. The perovskite layer, as a semiconductor material, functions as the absorption layer responsible for generating charges, while the ETL and HTL layers are used to extract and transport the photogenerated carriers. In a typical PSC stack structure, the perovskite layer lies between ETL and HTL, and the morphology and properties of the ETL/HTL film layer will directly affect the deposition quality of the perovskite layer [12]. Therefore, the film quality of ETL/HTL has become an essential driving factor for developing efficient and stable PSCs.

Film measurement is an integral part of the commercial production and scientific research of PSCs, and it guarantees the quality of each film layer. Optical measurement, with its non-contact, non-destructive, and pollution-free characteristics, is widely used in various fields. Among them, the spectroscopic ellipsometry (SE) measurement technique is employed to detect the change in the polarization state after the interaction (transmission or reflection) between polarized light and the sample. Then, the sample’s geometric characteristics (e.g., thickness and roughness) and photoelectric characteristics (e.g., complex refractive index/complex dielectric function, photoconductivity, absorption coefficient/extinction coefficient, and bandgap) can be obtained. The quantitative characterization of the geometric and photoelectric properties of PSC films can benefit the evaluation of the films’ quality. SE is often used to achieve the non-destructive measurement of semi-infinite thick substrates and films, and it can simultaneously obtain multiple measurement parameters. Hence, SE can obtain more sample information than traditional optical measurement techniques in one measurement [13,14].

**Figure 1:**
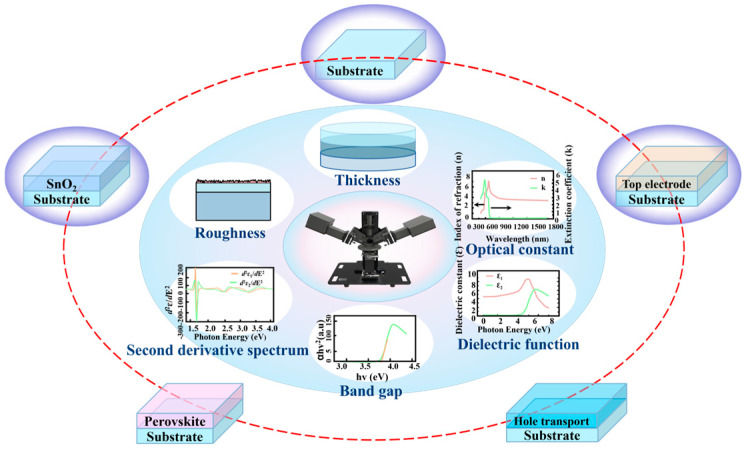
Measurement and characterization of photoelectric and geometric properties for each film layer of a typical SnO_2_-based PSC by SE.

SE is advantageous in that it is non-destructive, non-contact, and high-precision (thickness sensitivity: ~0.1 Å). SE is highly sensitive to changes in the properties of film materials; as such, it can be used to reveal the influence of some factors on the properties of the films of PSCs. It has become a powerful tool for the measurement and characterization of nanomaterials, which can help develop and optimize PSCs [15]. As shown in Figure 1, each film layer of PSCs with a smooth surface is prepared on a standard substrate. Then, the geometric and photoelectric characteristics of each film layer of PSCs can be accurately obtained using SE. Based on the characterization of each film layer, the characteristics of PSC multilayer films can be accomplished. After obtaining the optical constants of films, the optical and electrical properties of PSC devices can be further simulated.

The article aims to complete a review of the current literature describing the measurement and characterization of the photoelectric and geometric properties of PSC film layer materials using SE. Firstly, the influencing factors of the film quality and the spectroscopic ellipsometry measurement technique are described. Subsequently, the application of SE in characterizing film layer materials in PSCs is introduced. The advantages of SnO_2_ in PSCs as the ETL layer are presented, and the characterization of film layer materials in PSCs using SE is comprehensively summarized. Then, the application of SE combined with various measurement techniques in the measurement of the photoelectric and geometric properties of PSC films is discussed. Finally, the future development of SE measurement technology in the application of PSCs is prospected.

## 2. Perovskite Solar Cells and Spectroscopic Ellipsometry

**Figure 2:**
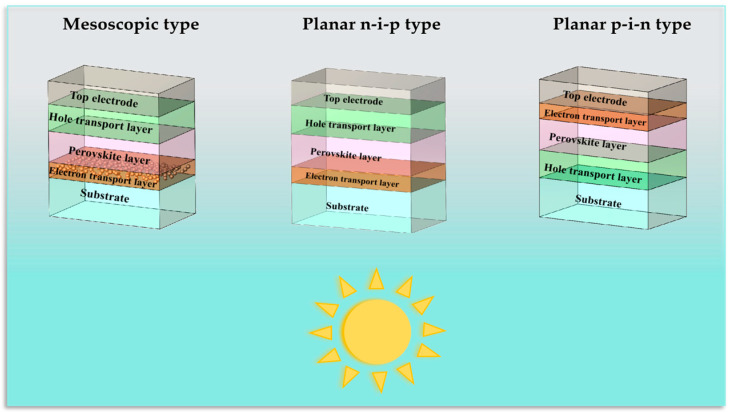
Three common structures of PSCs.

Three common structures of PSCs are shown in Figure 2, as follows: mesoscopic n-i-p type, planar n-i-p type, and planar p-i-n type, respectively. Here, n, i, and p represent the electron transport layer, perovskite layer, and hole transport layer, respectively. Whether the planar n-i-p or planar p-i-n type, the perovskite layer is between the electron transport layer and the hole transport layer. The difference is whether the electron or hole transport layer is deposited on the substrate. The light passing through the electron transport layer to reach the perovskite layer is the n-i-p type; whereas, the light passing through the hole transport layer to reach the perovskite layer is the p-i-n type [16,17].

### 2.1. Influencing Factors of Film Quality

**Figure 3:**
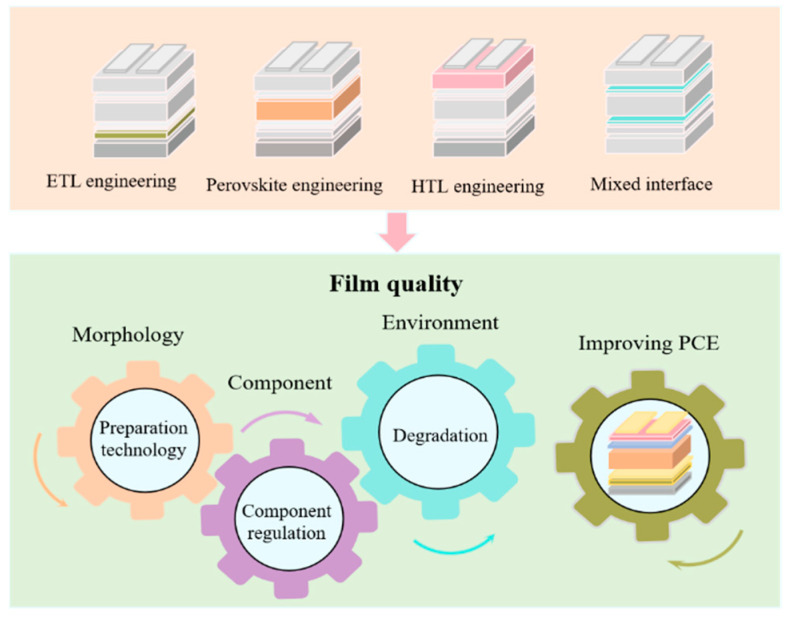
Influencing factors of power conversion efficiency of PSCs.

Power conversion efficiency and device stability are the two most critical challenges for PSCs. As shown in Figure 3, scholars have carried out much research on ETL engineering, perovskite engineering, HTL engineering and mixed interface layers to improve PSC efficiency and resist the external environment’s influence. Film quality plays a significant role based on the above technologies [18,19].

Film quality can be affected by several factors (e.g., morphology, component, and environment). The morphology of the film is directly affected by some technologies, such as different preparation techniques and annealing temperatures. During the preparation of each film layer, composition regulation can directly affect the optical properties of films. The optical properties of films can be directly changed by anion or cationic doping, which can affect the photoelectric properties of PSCs. As is well known, the perovskite layer is unstable in PSCs. The perovskite layer will degrade when the external environment changes (e.g., temperature, humidity, and light), which can affect the properties of films. When there is a roughness layer between two film layers or a roughness layer between a substrate and a film layer caused by a non-parallel interface, the device’s optical loss and electrical loss will be generated. In a laminated construction, performance is affected by the device’s mixed interface layers, which is an essential factor in developing PSCs with better performance [20].

High-coverage and uniform films are critical factors in obtaining high-efficiency PSC devices. The geometrical and photoelectric properties of films can reflect the quality of films to a certain extent. Firstly, the accurate acquisition of the optical constant of the film layer, namely, complex refractive index(n, k)/complex dielectric constant (ε_1_, ε_2_), is essential for the design of PSCs. On the one hand, it provides basic physical parameters. On the other hand, it is related to various properties of samples, including morphology, crystal quality, chemical composition, and electrical conductivity [21]. Additionally, an accurate understanding of film layers’ optical constants can help simulate the performance of PSCs. Secondly, because the electronic transition is the basis of photovoltaics, it is vital to understand the extinction coefficient/absorption coefficient and optical bandgap of film layers in PSCs from which the potential efficiency and cost of PSCs can be inferred. The extinction coefficient/absorption coefficient can reflect the absorption capacity of the material. The optical bandgap can reflect the optical performance of the semiconductor, which determines the required minimum energy to excite the semiconductor. Finally, it is necessary to consider the geometric characteristics of films (i.e., the film thickness and surface roughness). The ideal thickness of the film layer can balance the contradiction between the absorptivity and the carrier transport distance, which is advantageous to the extraction and transport of the photogenic carrier [22]. The surface roughness of the film can cause light scattering, and then, it can affect the photoelectric characteristics, such as the refractive index, extinction coefficient, and resistivity.

### 2.2. Spectroscopic Ellipsometry Measurement Technique

**Figure 4:**
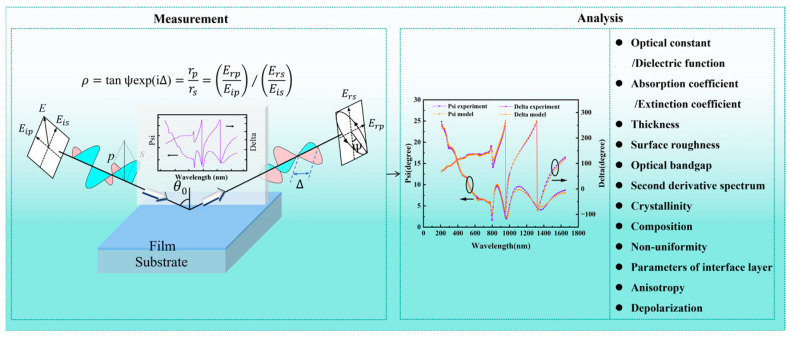
The basic principle of SE measurement and analysis.

SE measurement technology, as an indirect measurement technology, requires a process of measurement and analysis, as shown in Figure 4. Based on the measurement of the polarization state of light, SE can probe the changing information of the polarization state after the interaction between the light and sample; following that, two ellipsometry parameters (amplitude ratio Ψ/phase difference Δ) can be obtained. After that, suitable structural and optical models are established based on the Fresnel equation to obtain model data that fits with the measured data, from which the characteristics of the sample (geometric and photoelectric characteristics) are obtained [23]. In addition, SE can obtain some information, such as crystallinity, composition, interface layer, and non-uniformity. Compared to traditional ellipsometers, the Mueller matrix ellipsometry (MME) measurement technique can obtain 16 elements of the Mueller matrix by changing three conditions: wavelength, incident angle, and azimuth angle. It can offer more information about the sample, such as anisotropy and depolarization, which can provide possibilities for the characterization of complex samples [24]. To obtain more accurate results, the SE measurements of a film at the same point are made at several different beam incidence angles, for example, 65, 70, and 75 degrees, with the subsequent simultaneous approximation of the results obtained. This provides additional information, which leads to a significant increase in the reliability of determining the parameters of the film under study. In addition, wider or other wavelength ranges are used instead of the standard ones from 200–400 nm to 800–1000 nm.The data analysis of SE generally includes two processes: forward modelling and reverse reconstruction. Two processes mainly involve four aspects: (1) SE measures the samples to obtain amplitude ratio Ψ and phase difference Δ. (2) The corresponding structural and optical models are set up to describe samples. (3) Fitting the measured data and data of the constructed model of samples. (4) The effectiveness of the fitting is evaluated.

In the process of SE data analysis, the construction of a sample’s optical model is an essential step. A suitable optical model will be established according to the ellipsometry data and prior knowledge. The geometric model and optical function model constitute the optical model of the film layer. Then, the measured data by SE and the generated data by the optical model are matched using an iterative fit algorithm over the entire spectral range. In general, the iterative fitting process is completed by the nonlinear Levenberg–Marquardt (LM) algorithm [25,26,27]. Usually, the fit results are evaluated by using the mean square error (MSE) [28,29].

PSCs are a complex multilayer film stack structure, and the entire device can be equivalent to stacked multiple optical models of single-layer film, which is conducive to the characterization of film properties by SE. Due to dispersive properties, the optical constant of the film varies with the wavelength of the incident light. Fortunately, the film’s thickness does not depend on the wavelength change, which provides convenience for SE analysis. The dispersion relationship of wavelength-dependent films can be described by point-to-point, B-spline, and dispersion models. Dispersion models include Cauchy, Sellmeier, Lorentz, Tauc–Lorentz, Gaussian models, and so on [30].

In ellipsometry characterization, it is necessary to know the properties of materials in advance to select a suitable dispersion model for fitting. For the modelling of materials without absorption, the Cauchy model or the Sellmeier model can be used. The thickness and refractive index of the sample can be easily obtained by the Cauchy model due to the few parameters required to fit. The Cauchy–Urbach model is used to describe the optical behavior of materials in the transparent and weakly absorbing regions. For semi-absorbent materials, the refractive index curve of samples can be described by the Cauchy model in the non-absorption spectral region, and then, the initial thickness value of the film can be obtained by fitting, after that, which can be replaced by the universal oscillator model. The universal oscillator model can be used to absorb materials. When the material has complex absorption properties, multiple Tauc–Lorentz and Gaussian can be used to match complex dispersion shapes. The Tauc–Lorentz model can extract some information of absorption behavior and energy gap. Tauc–Lorentz combined with the Lorentz or Drude–Lorentz models can provide more physical parameters (such as carrier behavior). The Gaussian model is used to describe the absorption properties of the material, especially in the local oscillation or absorption peak region of the material. Gaussian models are often combined with other models, such as the Lorentz or Tauc–Lorentz model.

**Figure 5:**
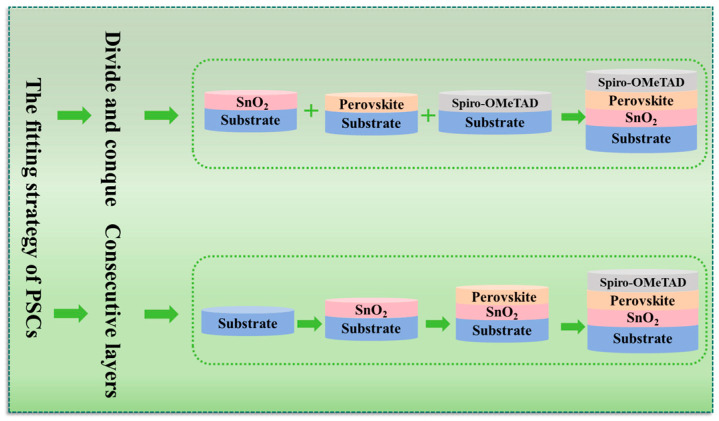
Fitting strategies of PSC multilayer films stack structure.

On a standard substrate, such as sapphire and glass, the modelling of a single film layer with a smooth surface is simple. However, in a multilayer film stack structure, the modelling of the sample will become more complex. Figure 5 shows the ellipsometry fitting strategy of the PSCs multilayer film stacked structure, and it includes two fitting strategies: “divide and conquer” and “consecutive layers”. If all parameters of all single layers are known, the parameters of the entire device can be measured based on the “divide and conquer” method. Another strategy for multilayer is termed the “consecutive layers” method. If parameters of a single layer are known, the parameters of multilayer film can be measured; after that, the parameters of the entire device can be obtained.

When there is an uneven surface of the film layer or mutual penetration between the two films, and the condition is met D(λ) < 1/2λ, D and λ are the dimension of microstructures and the wavelength of light probe, respectively. An effective medium approximation (EMA) model is usually used for modelling. Notably, when the thickness of films is thin, especially when the category of ultra-thin layer is reached, the refractive index and thickness of the film become strongly correlated. Therefore, simultaneously obtaining the thickness and refractive index using SE will be challenging. To address this limitation, Gu et al. proposed a complete analytical method of second-order Taylor expansion to determine the complex refractive index of ultra-thin film materials [31]. Nestler et al. used the first and second ellipsometry moments to simultaneously determine the ultra-thin films’ thickness and refractive index down to 5 nm thickness [32]. Li et al. used the thick film’s optical constant to fix the thin film’s optical constant to obtain the thin film’s thickness [33].

## 3. Measurement and Characterization of Perovskite Solar Cells by Spectroscopic Ellipsometry

### 3.1. Substrate

The substrate is located at the lowest end of a film photovoltaic device, and the light enters from the substrate. Usually, the substrate is a transparent glass covered with a transparent conductive film layer. The substrate with high optical transmittance can maximize the entry of external light into the device, which ultimately affects the photoelectric conversion efficiency. On the premise of using SE to characterize other film layers of film photovoltaic devices, it is necessary to first determine the optical properties of the substrate. In the data analysis of SE, the Cauchy and Sellmeier models can usually be used to describe the dispersion model of transparent, non-absorbing substrates. The Cauchy–Urbach model can be used to describe the dispersion model of slightly absorbing substrates in the ultraviolet spectral region.

When the substrate covered with TCO is used as the front electrode in PSC devices, the free carrier and interband transition will occur in the film, affecting the short circuit current. Interband transition occurs in the high-energy region, and free carrier absorption occurs in the lower-energy region. Therefore, the optical absorption of TCO can be described by superimposed interband transition and free carrier absorption. In SE data analysis, the Drude oscillator can be combined with an interband transition model, such as the Lorentz oscillator and Tauc–Lorentz oscillator, to describe the dielectric function of the TCO film layer. For instance, Stefano et al. studied the effect of annealing temperature on the optical properties and electrical properties of an indium-doped tin oxide (ITO) film using a Drude–Lorentz oscillator model [34].

### 3.2. Electron Transfer Layer (ETL)

In PSCs, ETL is another crucial film layer besides the perovskite layer. It can facilitate the collection and transport of photogenerated electrons to the corresponding electrode and block holes. Many materials, such as titanium dioxide (TiO_2_), C_60_ (C_60_/BCP or C_60_/SnO_2_), zinc oxide (ZnO), and tin dioxide (SnO_2_), can be used as ETL to transport electrons [35,36]. In n-i-p planar PSCs, TiO_2_ is the most widely used ETL material. Unfortunately, it has low electron mobility ($10^{-4}{\mathrm{cm}}^{2}\cdot V^{-1}\cdot s^{-1}$) [37] because of the existence of traps. Meanwhile, high-temperature sintering (>450 °C) [38] is required in the preparation process to improve crystallinity and carrier mobility. These factors limit the development of flexible PSCs. In recent years, n-type transparent semiconductor oxide SnO_2_ material has become an excellent ETL because of its excellent optical and electrical properties [39].

(1) SnO_2_ Materials: SnO_2_ material, as an electron transport layer, has very similar properties to TiO_2_, so it is an ideal material to replace TiO_2_. Currently, SnO_2_ used in PSCs has a tetragonal rutile structure. SnO_2_ ETL has better properties than TiO_2_ ETL, such as higher electron mobility (250 cm^2^V^−1^s^−1^), more suitable bandgap (3.6–4.31 eV) [40], more matched energy levels, and so on. Additionally, SnO_2_ ETL can be prepared at room temperature. These excellent properties can improve the electron extraction speed and transport efficiency from the perovskite layer. The recombination at the ETL/perovskite interface can be reduced, which will be a significant advantage in the large-scale commercialization process of flexible PSCs [41,42]. SnO_2_ film has absorption in the ultraviolet region, and the extinction coefficient is almost 0 in the visible and infrared regions. Based on these, it can enhance the light absorption of the perovskite layer and reduce the current loss [43].

(2) Effect of SnO_2_ Film Quality on PSCs: SnO_2_ films with good density, few pinholes, uniformity, and ultra-thinness are of great significance in improving the performance of PSCs. The uniform and dense film layer can ensure the smooth arrival of electrons from the perovskite layer to the substrate. Thereby, it can prevent electron reverse transport. Additionally, it can block holes to inhibit electron–hole recombination. In contrast, an uneven film surface will result in a large roughness, which affects the scattering of light and lead to inaccurate optical constants of films. The films with poor density may have some interstices between the ETL–perovskite or the ETL–substrate, which can result in optical and electrical losses. To ensure high light transmittance, ETL needs a suitable thickness. ETL with extremely thin thickness may lead to direct contact between the perovskite layer and substrate, which is unable to carry out effective electron transport. ETL with thicker thickness may increase the possibility of electron–hole recombination and then reduce light transmittance, which can deteriorate PSC performance. In PSCs, the thickness of common SnO_2_ ETL film is relatively thin, such as 15 nm [44], 25 nm [45], 30 nm [46], and 40 nm [47]. The electron transport characteristics of ETL with different thicknesses are different, which can result in different performance curves of photocurrent–photovoltage [48].

(3) Measurement and Characterization of SnO_2_ Materials Using SE: SnO_2_ films can be prepared by some technologies, such as chemical vapor deposition, sputtering, sol-gel, chemical spray pyrolysis, ion beam-assisted deposition, and so on [49,50]. Different film preparation techniques can result in changes in the properties of the film (e.g., optical constants). Some factors affecting the optical properties of the film (surface inhomogeneity, interface, and preparation method) can be monitored using SE. SE is not only very sensitive to the surface but also has a high sensitivity for the interface structure of films. SnO_2_ film is almost transparent in the visible spectral range. Thus, its wave-dependent refractive index curve can be described by the Cauchy model. The Cauchy model can determine the initial value of film thickness, and then, the full-band spectrum can be fitted by B-spline. Subsequently, the oscillator model is parameterized. EMA model is used to fit the roughness of films.

**Figure 6:**
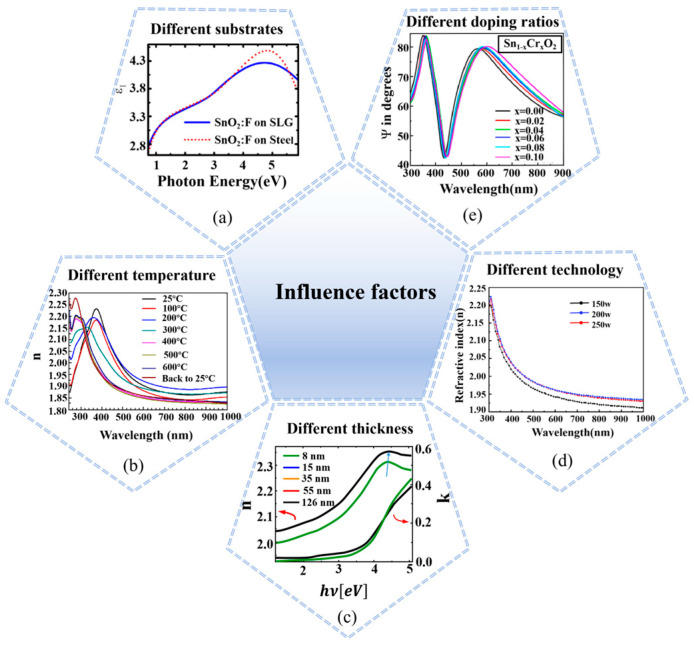
Characterization of SnO_2_ films by SE. (**a**) Different substrates [54]. (**b**) Different temperature [53]. (**c**) Different thickness [51]. (**d**) Different technology [52]. (**e**) Different doping ratios [55].

Figure 6 depicts the characterization results of SnO_2_ films by SE. It can be observed from the figure that the optical constant/dielectric function of the films will change with some control factors (e.g., substrates, proportion of ion doping, preparation process, different temperatures, and film stress caused by different thicknesses) change. Rus et al. analyzed that the optical constant and optical bandgap would change with the film thickness increase due to the film thickness change’s strain effect [51]. Shanker et al. used SE measurement technology to show that the refractive index of films is different under different preparation processes [52]. Gong et al. presented that temperature could affect the optical properties of films [53]. The relevant literature is summarized in Table 1. The optical constants not only determine the film’s transmission, reflection, refraction, and absorption properties but also can be used to study the band structure and optical transition. Accurately measuring the thickness and optical constants of the film layer is crucial for the design and optimization of PSCs. Additionally, the molecular orientation of SnO_2_ film may lead to optical anisotropy, and the optical anisotropy of SnO_2_ film can be accurately characterized by multi-incidence ellipsometry.

(4) Ion Doping of SnO_2_ Film: Proper doping can increase the carrier concentration, reduce the charge recombination at the interface, and improve the electron mobility, which can facilitate the rapid extraction of electrons from the perovskite layer. The most common doped SnO_2_ is ITO and fluorine-doped tin oxide (FTO) deposited on the glass substrate. As a non-toxic and chemically stable material, FTO is often used in transparent conductive layers. Adding fluorine increases the free carrier concentration and conductivity of SnO_2_ films [56]. Metal doping is an effective method to reduce the trap state and improve electron extraction. Many metals can be used to change the conductivity of SnO_2_ films [57,58,59] such as molybdenum, cesium, gallium, and lithium. In addition, some metal oxides can form complex bilayers with SnO_2_ to improve the efficiency of PSCs [60]. The effect of ion doping on the optical properties of films, such as complex refractive index/complex dielectric function and optical bandgap, can be measured by SE. 

**Figure 7:**
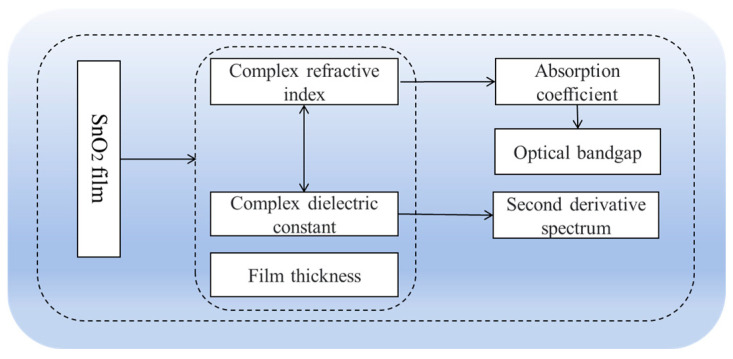
Analysis procedure of geometric and photoelectric properties of SnO_2_ films by SE.

Figure 7 illustrates the SE analysis process of the geometric and photoelectric characteristics of SnO_2_ films. The films’ optical constant/dielectric function and thickness can be obtained by the Ψ and Δ spectra. The relationship between the absorption coefficient and extinction coefficient satisfies α=4πk/λ, where α is the absorption coefficient, k is the extinction coefficient, and λ is the wavelength [61]. The optical bandgap of SnO_2_ can be calculated using the Tauc plot method, which is obtained by the following relation: $\left(\alpha hv\right)=B{\left(hv-E_{g}\right)}^{n}$, where $E_{g}$ represents the optical bandgap, B is the constant, and hv is the photon energy. The intercept of the linear fitting line extrapolated from the energy axis is the optical bandgap of the film [61]. Additionally, when the optical bandgap of the film is included in the dispersion model, the bandgap value of the material can be directly obtained. The second derivative spectrum of the material is usually related to the band structure of the crystal, and it involves the information of the electronic transition; hence, it can be used to determine the energy value of the optical transition and perform critical point analysis.

**Table 1:** Measurement and characterization of SnO_2_ material by spectroscopic ellipsometry.

Materials	Spectral Range	Incidence Angle (°)	Oscillator Model	Roughness (nm)	Film Thickness (nm)	Bandgap (eV)	Ref.
SnO_2_	191–989 nm	60, 65, 70	3Tauc–Lorentz	0.2, 0.7, 1.8 (AFM)	/	3.90–4.35	[62]
SnO_2_	245–1690 nm	60, 65	3Lorentz	/	123.59, 156.89, 97.88	/	[63]
SnO_2_	400–1800 nm	70, 75	/	8.01, 19.87 (SE)	110.16, 135.99 (SE)	3.6, 3.8 (SE)	[64]
SnO_2_	1.2–5.0 eV	65, 70, 75	B-spline	0.10, 0.21 (AFM)	15, 126 (AFM)	4.0–4.25	[51]
SnO_2_	400–1700 nm	65, 75	Cauchy–Urbach	0.59, 0.48, 0.11, 0.35 (AFM)	196.1, 180.4, 178.0 151.8 (SEM), 203.6 (SE)	/	[65]
SnO_2_	300–1200 nm	70	Lorentz	9.9–27.7 (AFM)	150–350 (SE)	3.98–4.09	[66]
SnO_2_	300–1000 nm	55, 65, 75	Lorentz	/	256.6–496.8 255.1–537 (SE)	3.69, 3.83	[52]
SnO_2_	350–1100 nm	65, 75	Cauchy–Urbach	9.4	166.3	3.2	[67]
SnO_2_	300–1000 nm	75	B-spline	0.97–1.40 (AFM)	390–472 (SE)	/	[53]
SnO_2_	1.46–6.2 eV	60	Tauc–Lorentz	/	30.2, 36.5 (SE)	/	[68]
SnO_2_	200–1000 nm	/	/	3.9 (AFM)	/	3.8–4.4	[69]
SnO_2_	300–1700 nm	50, 60, 70	Tauc–Lorentz Drude	/	4	/	[70]
SnO_2_:Fe	300–800 nm	70	Leng–Lorentz	/	/	3.44–3.58 (SE)	[71]
SnO_2_:F	0.035–5.89 eV 0.75–5.89 eV	/	Drude Tauc–Lorentz	3.3, 1.4 (SE)	393, 111.9 (SE)	3.45, 3.6 (SE)	[54]
SnO_2_:F	300–1700 nm	65	Lorentz–Lorentz	/	338–756 (SE)	3.7, 4.7 (SE)	[72]
SnO_2_:Sb	0.7–6 eV	65, 70, 75	Drude	/	100	4.079	[73]
SnO_2_:Cr	500–900 nm	60, 65, 70, 75	B-spline	3.45, 6.52 (SE)	151, 155 (SE)	/	[55]

The literature on the measurement and characterization of SnO_2_ films and doped SnO_2_ films by SE is summarized in Table 1, including film materials, spectral range, incidence angle, oscillator model, roughness, film thickness, and bandgap. From Table 1, the common oscillator model can be obtained, which will help to fit the SnO_2_ film. It is worth noting that some literature uses different oscillator models to fit. Appropriate models will be selected based on specific research objectives.

In the ellipsometry fitting, it is necessary to know the properties of SnO_2_ materials in advance to select a suitable dispersion model for fitting. The range of bandgap of tin oxide (SnO_2_) is about 3.6–4.31 eV; hence, when λ≥300, the Tauc–Lorentz and Lorentz models are usually used in the ellipsometry fitting. However, when λ≥350 or λ≥400, there is no absorption, or there is slight absorption in the selected wavelength range. Usually, the Cauchy–Urbach model is used in the ellipsometry fitting. The Cauchy–Urbach model can determine the thickness and refractive index of the sample. The Tauc–Lorentz model can obtain more information about the sample, such as absorption behavior and energy gap information. The optical properties of the doped SnO_2_ film will change, therefore, it is crucial to select appropriate oscillator models to match the measurement data.

### 3.3. Perovskite Layer

**Figure 8:**
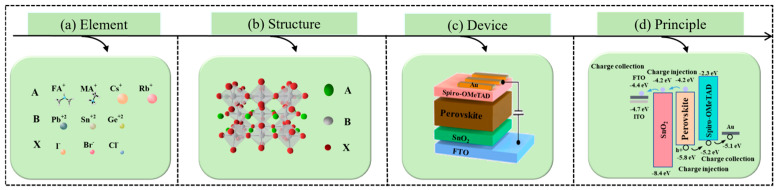
Perovskite materials and devices. (**a**) Element [16]. (**b**) Structure [75]. (**c**) Device [76]. (**d**) Principle [77].

Figure 8 displays the ion species, crystal structure, a typical SnO_2_-based perovskite solar device, and the function of each film layer. The chemical formula of perovskite materials satisfies ABX_3_. A, B, and X sites can accommodate a large number of types. Thus, it can be said that the types of perovskite materials are vast. The ideal structure of perovskites is cubic crystal. Herein, A is a cation (e.g., MA^+^, FA^+^, and Cs^+^), B is a cation (e.g., Pb^2+^ and Sn^2+^), and X is an anion (e.g., I^−^, Cl^−^, and Br^−^) [74].

Perovskite materials are used to generate charge in PSCs. When light is irradiated to the surface of the perovskite layer, the photon energy is greater than the bandgap width of the perovskite material, and the material will absorb photons to generate electron–hole pairs. Then, the electron and hole will migrate to the corresponding electrode under the effect of the internal electric field, which can be transmitted through each film layer and the mixed interface layer.

(1) Effect Factors of Perovskite Film Quality: There are some factors that can affect the properties of perovskite films, such as composition, structure, thickness, surface morphology, preparation method, and external environment. Further, the photoelectric performance of PSCs will change. It is worth mentioning that the thickness of the perovskite film layer located between the ETL and HTL is usually about 300–600 nm better to extract electrons and holes to the corresponding electrode. In total, the film quality of the perovskite layer is the key factor in obtaining high-performance solar cell devices.

(2) Roughness of Perovskite Film: The output power of PSCs is mainly determined by two factors, i.e., $J_{SC}$ and $V_{OC}$; its corresponding mathematical expression can be written as P∝Jsc×Voc. The absorption spectrum can calculate these two important parameters of solar cells (i.e., $J_{SC}$ and $V_{OC}$); that is, the final potential of PSCs can be judged from the absorption spectrum of the perovskite layer. The absorption spectrum of the film can be obtained by SE. First, the extinction coefficient k can be obtained by fitting the measured Ψ spectrum and Δ spectrum by SE. After that, the absorption spectrum can be generated by the formula α=4πk/λ. It is important to note that the roughness of films can affect the performance of the PSCs. Usually, to improve the short-circuit current density of PSCs, the perovskite layer needs to have an appropriate roughness. However, when films have a large roughness, the degree of light scattering will be increased, which is to the disadvantage of determining the optical properties of films by SE. If the optical response of the rough surface of films is not expressed correctly, the absorption coefficient of films will be overestimated. In this case, PSCs’ external quantum efficiency (EQE) will be overestimated.

**Figure 9:**
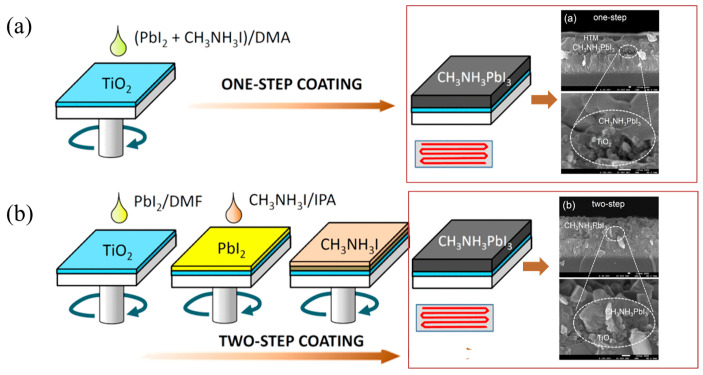
Preparation process of perovskite film [80]. (**a**) One-step coating. (**b**) Two-step coating.

There are some common preparation methods for perovskite films, such as the one-step solution, two-step solution, and gas phase deposition. Different preparation methods will show different forms of perovskite films, which can affect the properties of perovskite films [78,79]. Figure 9 illustrates the preparation methods of perovskite film with one-step and two-step solutions. It is noteworthy that films will present different surface topography using different methods. Jeong-Hyeok et al. found that the pores are completely filled with perovskite by using the two-step coating different from that prepared by the one-step method, which can avoid direct contact between ETL and HTL [80]. It is important to note that the surface roughness of perovskite films prepared by the solution process is unavoidable. In SE fitting processing, when the roughness of films meets the conditions of the EMA model, the roughness of films can be fitted by the EMA model. The EMA model describes the roughness of the entire film surface. On the other hand, the relationship between the roughness obtained by atomic force microscopy (AFM) and the roughness obtained by SE is obtained by the following [81]: $d_{s}\left(SE\right)\approx 1.5d_{rms}\left(AFM\right)+4\overset{\circ }{A}$, where $d_{s}\left(SE\right)$ represents the roughness obtained by SE, and $d_{rms}\left(AFM\right)$ is the root-mean-square roughness obtained by AFM. There is an apparent linear relationship between the two measurement results; therefore, it is a method for SE analysis that refers to the values of measured roughness by AFM.

(3) Fitting Strategy of Perovskite Films: Perovskite materials have a prominent bandgap characteristic. They are transparent in the infrared spectral region and have absorption in the visible spectral region. In the ultraviolet region, the complex absorption characteristics of films can be analyzed. Polycrystalline perovskite films exhibit optical isotropy; therefore, the measurand two parameter curves (Psi and Delta) by SE are sufficient to characterize the thickness and optical constant of the perovskite films.

In the process of SE fitting, the initial thickness of the film can be determined by the Cauchy dispersion model in the infrared visible region. Then, a B-spline can be used to fit the full-band spectrum with the appropriate step size, and the fitting curve can be obtained initially. Finally, the B-spline fitting result can be characterized by using appropriate oscillator models to obtain the film’s final fitting curve. At the same time, it is necessary to consider the surface roughness, transition layer, and porosity. Usually, the complex dielectric functions of perovskite films are characterized using multiple Tauc–Lorentz oscillator models. Tauc–Lorentz is suited for absorbing materials in PSCs, because it effectively models complex refractive index behavior across the spectrum, and it can also extract optical bandgap information.

**Figure 10:**
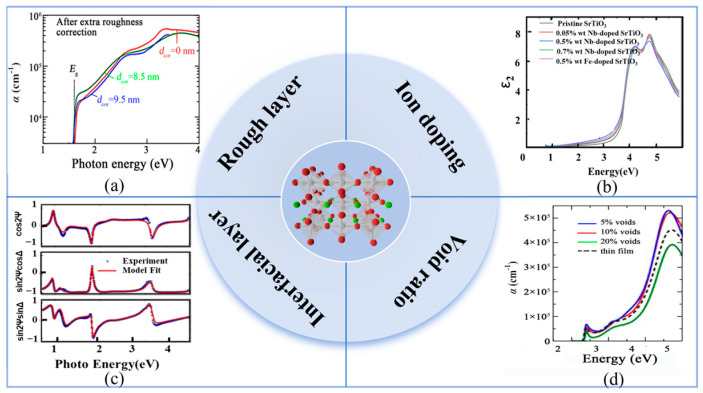
Influence factors of perovskite films. (**a**) Rough layer [82,83]. (**b**) Ion doping [86]. (**c**) Interfacial layer [20]. (**d**) Void ratio [61].

(4) Characterization of Geometric and Photoelectric Properties of Perovskite Films: Many studies have confirmed that SE is sensitive to the change in perovskite film properties. Hence, to reveal the effects of some factors (e.g., rough layer, void ratio, interfacial layer, and ion doping) on the optical properties of perovskite films, the SE measurement technique is used to monitor the perovskite films. As depicted in Figure 10, Fujiwara et al. investigated the effect of the rough surface of the film on the SE analysis results and further analyzed the absorption spectra of rough and un-rough films [82]. When the surface roughness analysis of films is not correct, there is a higher α value. Hence, SE is sensitive to the surface structure of films, and the correct modelling of the surface roughness is a key factor for obtaining accurate optical parameters. Alias et al. proved that different void volume fractions would affect the refractive index and absorption coefficient of films. Therefore, it is necessary to consider the effect of void proportion in SE data analysis [61,83]. Subedi et al. confirmed that the physical mixed interface layer between two film layers could influence device performance. When the interface layer is added, not only is the spectral curve better fitted, but the results are also more accurate [20]. Ion doping can also affect the optical properties of perovskite films. Ndione et al. explored the change in the properties of perovskite films with the increase in Br ion concentration [84]. EI-Naggar et al. doped Cs ions into FA_0.95_MA_0.05_Pb (Br_0.02_I_0.98_)_3_(CsI)_x_ (x = 0.02, 0.05, 0.07) to explore the effect of ion doping on perovskite films; at the same time, they further discussed the effect of doping on device performance [85]. Zhang et al. found that extra momentum was provided to enable indirect absorption by doping SrTiO_3_ with Nb elements from 0.05 to 0.7% wt. [86].

**Figure 11:**
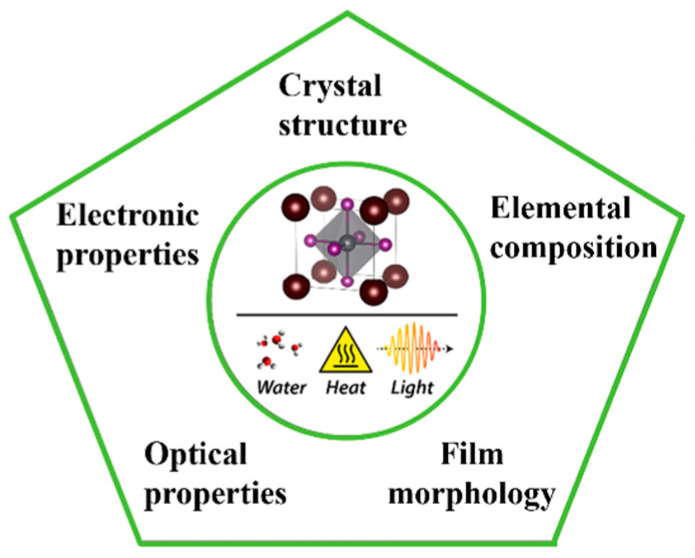
Influence of the external environment on perovskite films [87].

Perovskite materials are susceptible to degradation by the external environment (e.g., humidity, heat, and light) and further affect the properties of films, as depicted in Figure 11. Hence, it is important to understand the influence of environmental factors on the properties of perovskite films. It is noteworthy to highlight recent studies revealing the environmental influence of perovskite materials using SE.

**Figure 12:**
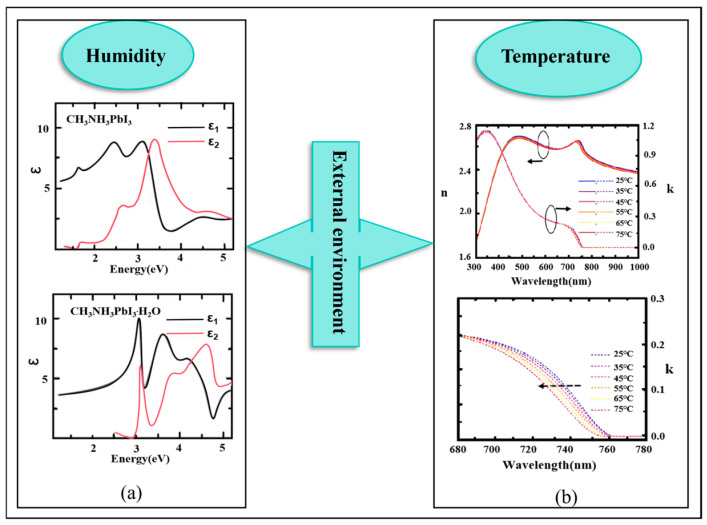
Influence of external environment on the properties of the perovskite film characterized by SE. (**a**) Humidity [89]. (**b**) Temperature [90].

Kundu et al. introduced in situ measurement techniques to track the degradation of perovskite films or devices throughout their life cycle. Jiang et al. used SE to measure the temperature-dependent optical properties of CH_3_NH_3_PbI_3_ over the temperature range from 77 K to 297 K. They found that the dielectric function increases with decreasing temperature [88]. Illustrated in Figure 12, Leguy et al. considered humidity a primary influencing factor in exploring the changes in optical properties with the degradation of perovskite materials. In humid air, the CH_3_NH_3_PbI_3_ film would generate the hydrated phase CH_3_NH_3_PbI_3_**^.^**H_2_O during the degradation process, and the dielectric function changed significantly [89]. In the temperature range of 25–75 °C, Raja et al. studied the variation trend of the optical constants of ternary cationic perovskite films, and they explored the influence of high-temperature conditions on the properties of perovskite films. Further, they simulated the optical characteristics of PSCs [90]. Table 2 summarizes some literature on the measurement and characterization of perovskite materials in recent years by SE, including film material, spectral range, incidence angle, oscillator model, roughness, thickness, and bandgap. The surface roughness of all the perovskite layers is based on the EMA model. In reference [91], perovskite is the single crystal, while other references are films. From Table 2, the common oscillator model of perovskite films can be obtained, and some characteristics of the perovskite films can be observed. Usually, the models of perovskite films are fitted by several Tauc–Lorentz (TL) models.

### 3.4. Hole Transport Layer (HTL)

As a p-type semiconductor material, HTL has a high transfer rate for holes. It can block electron transport and promote the collection and transfer of holes to the corresponding electrode. HTL materials can be divided into organic, e.g., Spiro-OMeTAD (2,2′,7,7′-tetrakis(N,N-di-p-methoxyphenylamino)-9,9′-spirobifluorene), PEDOT: PSS (Poly(3,4-ethylenedioxythiophene):polystyrene sulfonate), and PTAA (poly[bis(4-phenyl)(2,4,6-trimethylphenyl)amine]), and inorganic, e.g., CuI, Cu_2_O, CuO, and NiOx. Spiro-OMeTAD is commonly used for n-i-p structures [104]. PEDOT: PSS and NiOx are widely used for p-i-n trans structures. PEDOT: PSS film is widely accepted as optically anisotropic [105]. Notice that most of the organic semiconductors HTL materials used for PSCs have low thermal stability. For instance, Spiro-OMeTAD is an excellent semiconductor material, but its thermal stability is poor. There are several reasons for its instability. At high temperatures, the ions of metal electrodes can diffuse into the Spiro-OMeTAD film layer. On the other hand, the Spiro-OMeTAD can generate phase transition and crystallization. Hence, these shortcomings limit the lifetime of PSCs [106]. Similarly, PTAA, as an organic HTL, also has low thermal stability. The p-type inorganic semiconductor NiOx has a bandgap range of 2.5–3.8 eV, and it also has some outstanding characteristics of high transparency and high chemical stability [107]. Kim et al. introduced the thermal stability of various hole transport layers [108].

**Figure 13:**
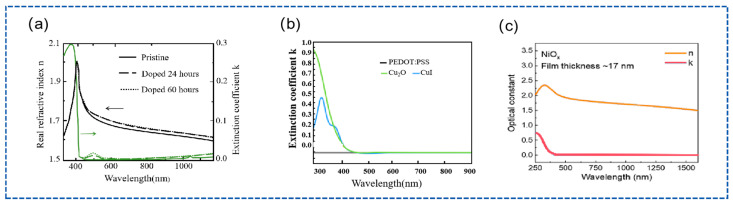
(**a**) Optical constant of the Spiro-OMeTAD film [113]. (**b**) Optical constant of PEDOT: PSS, Cu_2_O, and CuI films [114]. (**c**) Optical constant of the NiOx film [115].

(1) Characterization of Geometric and Photoelectric Properties of HTL: There are some standard preparation methods of HTL, such as sputtering, pulsed laser deposition, and atomic layer deposition [109,110,111]. Generally, HTL materials are highly transparent in the visible spectrum, and there is absorption in the ultraviolet region of the spectrum. The different HTL materials begin to absorb at different wavelengths in the ultraviolet region. Similar to the fitting strategy of the ETL, firstly, the thickness of the HTL materials can be fitted using the Cauchy model in the visible spectral region, and then, the optical constant of the film can be determined using the B-spline over the entire spectral range. Finally, the fitting curve obtained by the B-spline will be parameterized using oscillator models. The oscillator model can use the Tauc–Lorentz model, the Lorentz model, or a combination of the Tauc–Lorentz model and Drude model [112]. Illustrated in Figure 13, Eerden et al. explored the change in the optical constant of the Spiro-OMeTAD films exposed to oxygen at different times. With the increase in contact time, the optical constant of the film continuously changes [113]. Hasan et al. presented the extinction coefficient of various HTL materials using SE. It is apparent that different materials have different extinction coefficients [114]. Zhumagali et al. determined the optical constant of NiOx film with 17 nm [115]. Manzoor et al. indicated detailed the measurement information of p-i-n trans structure film layers using SE [70]. Shim et al. obtained a complex refractive index (refractive index n and extinction coefficient k) of NiO film with a thickness of 25 nm using SE. Further, it can be observed that k=0 exists in the visible spectral region and infrared spectral region of films, and absorption exists in the near ultraviolet spectral region. On the other hand, it can be observed in detail that the transmittance of glass/ITO and glass/ITO/NiO samples are different, which will affect device performance [116]. Table 3 summarizes some literature on the measurement and characterization of HTL materials by SE, including film material, spectral range, incidence angle, oscillator model, roughness, thickness, and bandgap. From Table 3, the common oscillator model of HTL materials can be observed. The NiOx film has a bandgap ranging from approximately 3.6 to 4.0 eV. In the wavelength range excluding the bandgap (λ > bandgap), where the material will not have absorption, the Cauchy model is typically used to fit the measurement data. Conversely, in the wavelength range that includes the bandgap, the Tauc–Lorentz and Lorentz models are commonly applied for data fitting.

### 3.5. Metal Electrode Layer

The role of the metal electrode layer in PSCs is to transport photogenerated carriers to the external circuit and promote the generation of the photocurrent. Common metal electrode materials include silver, aluminum, and gold. It is noteworthy that, when the metal film is thin enough that light can penetrate it, the obtained characterization parameters of the film by SE will be more than those of the measurement data generated by SE. Namely, it can obtain measurement ellipsometry data (Ψ and Δ). However, the three parameters, refractive index, extinction coefficient, and thickness, need to be determined simultaneously. Hence, multiple possible values may be due to the strong correlation between the thickness and optical constant. As a result, film thickness and optical constants cannot be accurately analyzed. Herein, it can break a strong correlation by increasing the measurement information or reducing the parameters of unknown samples, such as multi-sample analysis, in situ analysis, interference enhancement, and a combination of SE and transmission (SE + T). In the SE + T method, to calibrate the optical constant of the metal film, the metal film is requested to evaporate on a transparent substrate with a known optical constant [123]. Usually, metal films have a strong absorption. When the metal film is thick enough, it can be regarded as a pure substrate. Hilfiker et al. described in detail various measurement methods and fitting effects of absorbing films based on SE [124]. Gong et al. found that the optical constant of ultra-thin silver films largely depended on the film thickness. In the near-ultraviolet to infrared wavelength range, the wavelength-dependent refractive index and extinction coefficient of ultra-thin silver films with different thicknesses are shown [125]. In the energy range 1.4–5 eV, Sundari et al. analyzed the optical constant of silver at the temperature range from 300 K to 650 K with 50 K intervals using SE, and it clearly showed the temperature dependence of the dielectric function [126].

## 4. Assist Techniques of Spectroscopic Ellipsometry

Spectroscopic ellipsometry is an indirect measurement method. High-precision data, correct optical model, and appropriate analysis strategy are vital for fitting the results of SE [83]. The optical model constructed for analysis only represents the approximate structure of the sample. Even if a better fitting effect is achieved based on the established optical model, the analysis results may still contain errors. Hence, to better measure and characterize the film layer’s geometric and photoelectric characteristics in PSCs, it is necessary to combine SE with various measurement techniques. It not only provides a reasonable initial value for SE but also verifies the results of SE characterization. An initial estimate value close to the actual value can better fit Ψ and Δ data.

A scanning electron microscope (SEM) and step profiler can not only be used to provide film thickness in the process of ellipsometry fitting but also to verify the correctness of the film thickness obtained by SE fitting. The existence of film roughness can be predicted in advance using AFM and a white light interference microscope, which will help SE establish the correct model. An ultraviolet-visible spectrophotometer (UV-Vis) can be used to determine film absorption, and then, the bandgap of the semiconductor film can be calculated, which is beneficial for the verification of SE analysis results. X-ray diffraction (XRD) can determine the composition and crystallinity of films, which can further assist the model analysis of ellipsometry. Table 4 summarizes some techniques that can be used to measure the geometric characteristics and photoelectric characteristics of the film layers of PSCs, including the measurement technique, abbreviation, type, film property, and measurement feature. These techniques can assist SE to characterize the properties of films.

**Figure 14:**
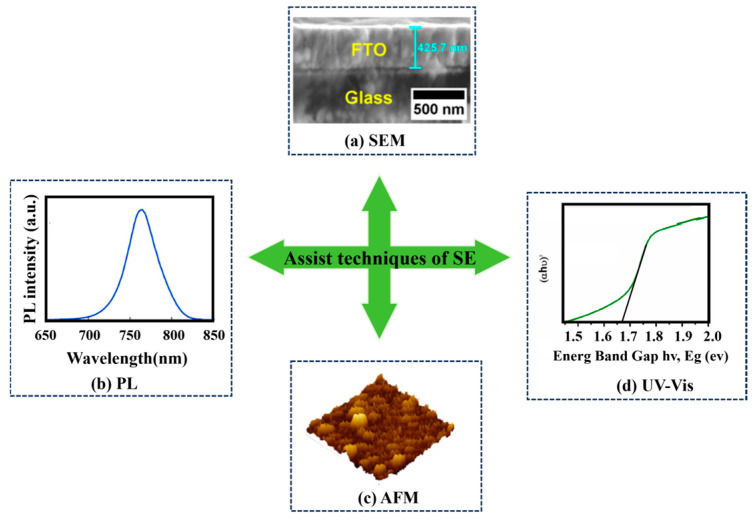
Assist technique in spectroscopic ellipsometry. (**a**) Scanning electron microscopy (SEM) [61]. (**b**) Photoluminescence (PL) [46]. (**c**) Atomic force microscopy (AFM) [61]. (**d**) Ultraviolet-visible spectroscopy (UV-Vis) [127].

As illustrated in Figure 14, Alias et al. characterized the thickness and roughness of FTO using SE, SEM, and AFM, and they found that SE is highly consistent with the results obtained from SEM and AFM; hence, the accuracy of ellipsometry characterization is verified [61]. Singh et al. measured the absorption spectrum of the CsPbI_3_ perovskite film using UV-Vis, and then, the optical bandgap of the perovskite film was obtained. Likewise, the optical bandgap can be obtained by SE. On the other hand, they measured the PL spectrum of the film, from which it can be observed that the emission peak of the film is concentrated at 760 nm [127]. Yan et al. measured the PL spectrum of perovskite films and obtained a peak value of 762 nm, which is consistent with the bandgap determined by the Tauc diagram [46]. Shirayama et al. used XRD to measure the composition of CH_3_NH_3_PbI_3_ film and expounded the effect of the annealing temperature on films. When the annealing temperature reached 100 °C, the CH_3_NH_3_PbI_3_ film could form the PbI_2_ phase, which provided a basis for SE modeling. SEM was used to show grain sizes at different annealing temperatures, and the surface morphologies of films could be observed. It is noteworthy that when D>0.1λ, the conditions of ellipsometry measurement would not be met. Therefore, the optical properties of films could not be measured using SE [83].

## 5. Future Outlook

As can be seen from the above, the SE measurement technique has been widely used to measure and characterize the geometric and photoelectric properties of film layer materials of PSCs. The parameter change with wavelength measured by SE is more interesting than any single measurement parameter obtained by other technologies, because SE can obtain multiple measurement parameters simultaneously. Based on characterizing the properties of monolayer films, SE can be used to predict the stack structure of multilayer films, including the stack thickness, total reflectance, and total transmittance, which provide key performance parameters for optimizing the device structure. The following aspects are worthy of attention from the current relevant research analysis.

(1) Development of a New Special Spectroscopic Ellipsometer: According to different working principles and uses, ellipsometers can be divided into different types, such as the visible light ellipsometer, infrared ellipsometer, single wavelength ellipsometer, and Muller matrix ellipsometer. Perovskite materials have good photoelectric properties; unfortunately, they are sensitive to the external environment. For instance, they are easily degraded by humidity, temperature, and light, which causes problems with the long-term stability and commercialization of PSCs. In general, to prevent perovskite materials from degrading when exposed to the environment, perovskite films are prepared in a glove box filled with nitrogen gas. Therefore, it is of practical value to develop a special ellipsometer that can not only be located in the glove box but also be suitable for monitoring the deposition quality of perovskite films.

(2) Reasonable Selection of Spectroscopic Ellipsometry Measurement Technique: The Muller matrix ellipsometer (measurable 4×4 order full Muller matrix) can obtain more information about a sample than the ordinary ellipsometer (two ellipsometer parameters amplitude ratio Ψ/phase difference Δ). The infrared spectroscopic ellipsometer can measure various infrared vibrations, including LO phonons and TO phonons. In the film deposition process, in situ ellipsometry measurement can be used to make multiple ellipsometry measurements of the same point on the sample; based on this way, the optical constant at a single wavelength corresponding to the thickness change with time can be obtained. For example, in situ ellipsometry measurement can track the dynamic variation of complex optical constants with thickness change during the preparation and degradation of perovskite films. In addition, in situ real-time ellipsometry measurement provides other independent dynamic analyses of sample properties. The variable incidence ellipsometry measurement technique is helpful in extracting the thickness and optical constants of each layer of PSC stack structure. In addition, more reliable results can be obtained from multi-sample analysis. For example, the optical constant can be determined using more than two samples of different thicknesses. Therefore, the reasonable selection of SE measurement techniques can monitor and control the deposition quality of PSC films in real time.

(3) Intelligent Development of Spectroscopic Ellipsometry Measurement Technology: With the rapid development of artificial intelligence, it is increasingly urgent to introduce machine learning and intelligent optimization algorithms into SE measurement technology. The research based on these will be a trend to promote the development of ellipsometry measurement technology in the future. In the data analysis process of traditional ellipsometry, it is necessary to select oscillator models to participate in the data fitting empirically. Also, reasonable initial values are needed to obtain accurate fitting parameters. Therefore, the results of the analysis vary from person to person. To ensure the accuracy of the analysis results, machine learning can be used to extract the feature information from ellipsometer data, and then, the oscillator model can be selected automatically to participate in the fitting process, which reduces the influence of human factors. On the other hand, the intelligent optimization algorithm is applied to data analysis, and the required parameters can be automatically fitted without similar initial values. For example, Ma et al. used the whale optimization algorithm (WOA) to fit the ellipsometry data and obtained better fitting results [128,129]. Patel et al. applied teaching–learning-based optimization (TLBO) to align model data with experimental data [130]. At the same time, machine learning can also be used to train the ellipsometer data to automatically obtain the film parameters (such as film thickness and optical constant), which has a faster solution speed. For example, Arunachalam et al. used machine learning to predict thickness [131]. Artificial intelligence can improve the efficiency of online ellipsometer monitoring, and it may become a hot spot in ellipsometer data analysis.

Therefore, as a non-destructive measurement technique, SE is necessary for the real-time detection of film deposition processes in different stages of PSC devices. In the future, advanced SE measurement technology will have a broader application in film photovoltaics.

## 6. Conclusions

The spectroscopic ellipsometry measurement technique has practical guiding significance in the research and development of film photovoltaics. It can extract the photoelectric and geometric characteristics of each film layer of PSCs and obtain the parameters of the whole device. The quantitative characterization of film layers is conducive to evaluating the deposition quality of films, which can further identify current loss mechanisms and predict the potential efficiency of PSCs. Then, the photoelectric conversion efficiency and stability of PSCs will be improved. This paper describes the advanced spectroscopic ellipsometry measurement technique and its application in the geometric and photoelectric properties of the film layers of perovskite solar cells. The characterization of perovskite materials is introduced in detail. Also, it paid attention to the advantages of SnO_2_ material as an electron transport layer in PSCs and the characterization of SnO_2_ material using SE. Further, the photoelectric parameters of the stacked structures of multilayer films can be obtained by characterizing the properties of the monolayer films. Based on this, SE measurement technology can be used to monitor and control the deposition quality of films in real time to meet the production requirements of PSCs from small-area to large-area module cells. Finally, it is pointed out that some worthy attention directions of the application of SE in PSCs are discussed. The development of new spectroscopic ellipsometers, the reasonable selection of ellipsometry measurement technology, and the intelligent acquisition and analysis of ellipsometry data may be the directions of future exploration in the hope that relevant researchers will collaborate to improve PSC efficiency and stability.

## Figures and Tables

**Table 2:** Measurement and characterization of perovskite materials by spectroscopic ellipsometry.

Material	Spectral Range	Incidence Angle (°)	Oscillator Model	Roughness (nm)	Thickness (nm)	Bandgap (eV)	Ref.	Year
CH_3_NH_3_PbI_3_	300–1700 nm	55, 60, 70	3Tauc–Lorentz	25 (AFM)	460.2 (SEM)	1.58 (SE)	[70]	2018
CsPbBr_3_	300–800 nm	75	7Tauc–Lorentz	37.43 (AFM)	800	2.39 (SE)	[92]	2018
CH_3_NH_3_PbI_3_	0.73–6.45 eV	65, 70, 75	Tauc–Lorentz	10 (SE), 13 (AFM)	446 (SE), 452	1.58 (SE)	[93]	2019
CH_3_NH_3_PbI_3_	245–1000 nm	60, 65, 70	Tauc–Lorentz	10 (AFM)	372 (SEM)	1.6 (SE)	[94]	2019
CsPbI_3_	300–1200 nm	43.9, 48.9, 53.9, 58.9, 63.9	Tauc–Lorentz, Gaussian	15 (AFM), 9 (SE)	468 (SE) 500 ± 25 (SEM)	1.69 (SE) (Tauc–plot)	[95]	2020
MAPb_1-x_Sn_x_I_3_ (x = 0, 0.4, 0.8, 1)	248–1240 nm	70	3Tauc–Lorentz	5.18, 8.89, 15.1, 19.2 (AFM)	96 ± 3 (SE)	1.54, 1.51, 1.49, 1.46	[96]	2020
MAPbBr_3_ (monocrystal)	245–1240 nm	60, 65, 70, 75	7Tauc–Lorentz	/	/	2.332 (SE)	[91]	2021
CsPbBr_3_	300–900 nm	55, 60, 65	1Tanguy, 2Lorentz	/	142.58	2.37 (Tauc–plot)	[97]	2021
Cs_0.05_(MA_0.17_FA_0.83_)_0.95_ Pb(Br_0.17_I_0.83_)_3_	200–2500 nm	55, 65, 75	2Tauc–Lorentz, 7Gaussian	20–30, 1–6 (SE)	400–550	/	[98]	2022
FA_0.95_MA_0.05_Pb (Br_0.02_I_0.98_)_3_(CsI)x (x = 0, 0.02, 0.05, 0.07)	/	70, 75	Tauc–Lorentz, Gaussian, Herzinger–Johs (HJ) Psemi-M_0_ parametric	5.18 (SE)	742.53 (SE)	1.63–1.72 (Tauc–plot)	[85]	2022
Cs_3_Sb_2×9_ (X = I/Br)	/	/	7Tauc–Lorentz	84 (AFM)	/	2.85	[99]	2023
MAPbI_3_	200–1000 nm	55, 65, 75	Several Tauc–Lorentz, Gaussian	/	/	1.57 (Tauc–plot), 1.5 (SE)	[100]	2023
MAPbI_3_	300–1000 nm	50, 60, 70	1Tauc–Lorentz, 3Gaussian	/	151	/	[101]	2024
FAPbI_3_/Cs_0.1_FA_0.9_PbI_3_	550–1000 nm	/	3Tauc–Lorentz	15, 23 /21, 15	374, 360 (SE) /382, 364 (SE)	/	[102]	2024
MAPbBr_3_	193–1690 nm	65, 70, 75	B-spline	/	/	2.35–2.46	[103]	2024

**Table 3:** The measurement and characterization of HTL materials by spectroscopic ellipsometry.

Material	Spectral Range	Incidence Angle (°)	Oscillator Model	Roughness (nm)	Thickness (nm)	Bandgap (eV)	Ref.	Year
NiOx	245–967 nm	70	Tauc–Lorentz and Drude	/	42.2, 40.0, 36.2 (SE)	/	[117]	2024
NiOx	350–1000 nm	50–70	Cauchy	1.32 (AFM)	42.6, 43.3., 48.4 (SE)	3.77	[118]	2023
NiOx	250–1000 nm	65–75	Lorentz	1.46, 0.46, 0.65, 1.35	9.5	/	[119]	2015
NiOx	0.735–5.887 eV	65	2Lorentz			3.66, 3.67, 3.69, 3.70		
NiOx	300–1100 nm	55, 65, 75	Tauc–Lorentz	/	20	/	[70]	2018
Spiro-OMeTAD	380–1200 nm	50, 60, 70	4 Lorentz and a Cauchy	/	100–300	/	[120]	2015
PEDOT: PSS	0.5–5 eV	70	Lorentz and Drude	10.3, 1.9 (AFM)	75 (SE)	/	[121]	2020
C60	250–1200 nm	50, 60, 70	3 Lorentz	/	15	/	[122]	2022

**Table 4:** SE combines multiple measurement techniques used for PSCs.

Techniques	Abbreviation	Type	Film Property	Feature
X-ray diffraction spectrum	XRD	X-ray	Crystallinity and grain size	The matter structure is determined according to the diffraction phenomenon
X-ray photoelectron spectroscopy	XPS	X-ray	Composition, content, and chemical valence state	Electrons with characteristic energy are collected to characterize the film components
Energy dispersive X-ray spectrum	EDX	X-ray	Film composition and content	Different elements have different characteristic X-rays
Photoluminescence spectrum	PL	Illuminant	Bandgap, carrier recombination, and extraction	Molecular luminescence is caused by absorption of light energy
Atomic force microscope	AFM	Probe	Surface morphology and thickness	The atomic force between the probe and the sample surface is detected
Scanning electron microscope	SEM	Electron beam	Surface, surface morphology, and thickness	The sample is scanned using a high-energy electron beam
White light interference microscope	/	Illuminant	Surface morphology	The principle of light interference is used
Transmission electron microscope	TEM	Electron beam	Surface morphology and thickness	The sample is scanned using a high-energy electron beam
Spectroscopic ellipsometry	SE	Illuminant	Geometric characteristics and photoelectric characteristics	Multiple characteristic parameters can be lossless characterized, simultaneously
Step profiler	/	Probe	Surface morphology and thickness	The probe moves over the film surface to measure the height difference for obtaining the film thickness
Surface profiler	/	Illuminant	Surface morphology	The interaction between light beams and matter is measured
Raman spectrometer	/	Laser	Chemical structure and stress	The light scattering technique is used
Ultraviolet visible spectrophotometer	UV-Vis	Illuminant	Absorption spectrum, reflection spectrum, transmission spectrum, and optical bandgap	The radiation intensity of absorbed/transmitted light of molecules or ions of the substance is measured

## Data Availability

Data are available upon request.

## Abbreviations

AFM	Atomic force microscopy
Cs^+^	Cesium
EDX	Energy dispersive X-ray spectrum
ETL	Electron transport layer
ESTI	European solar test installation
EMA	Effective medium approximation
EQE	External quantum efficiency
FTO	Fluorine-doped tin oxide
HTL	Hole transport layer
ITO	Indium-doped tin oxide
LM	Levenberg–Marquardt
MME	Mueller matrix ellipsometry
MSE	Mean square error
MA+	Methylammonium
FA+	Formamidinium
PSCs	Perovskite solar cells
PSC	Perovskite solar cell
PTAA	Poly[bis(4-phenyl)(2,4,6-trimethylphenyl)amine]
PEDOT: PSS	Poly(3,4-ethylenedioxythiophene):polystyrene sulfonate
SE	Spectroscopic ellipsometry
SnO_2_	Tin dioxide
Spiro-OMeTAD	2,2′,7,7′-tetrakis(N,N-di-p-methoxyphenylamino)-9,9′-spirobifluorene
SEM	Scanning electron microscope
TCO	Transparent conductive oxide
TiO_2_	Titanium dioxide
TLBO	Teaching–learning-based optimization
UV-Vis	Ultraviolet-visible
WOA	Whale optimization algorithm
XRD	X-ray diffraction
ZnO	Zinc oxide

## References

[1] Noh J.H., Im S.H., Heo J.H., Mandal T.N., Seok S.I. (2013). Chemical management for colorful, efficient, and stable inorganic-organic hybrid nanostructured solar cells. Nano Lett..

[2] Eperon G.E., Stranks S.D., Menelaou C., Johnston M.B. (2014). Formamidinium lead trihalide: A broadly tunable perovskite for efficient planar heterojunction solar cells. Energy. Environ. Sci..

[3] AlZoubi T., Mourched B., Gharram M.A., Makhadmeh G., Noqta O.A. (2023). Improving photovoltaic performance of hybrid organic-inorganic MAGeI_3_ perovskite solar cells via numerical optimization of carrier transport materials (HTLs/ETLs). Nanomaterials.

[4] Dong Q., Fang Y., Shao Y., Mulligan P., Qiu J., Cao L., Huang J. (2015). Electron-hole diffusion lengths>175 μm in solution-grown CH_3_NH_3_PbI_3_ single crystals. Science.

[5] Deng J., Jiang X., Liu Y., Zhao W., Tang G. (2022). Enhanced domain wall conductivity in photosensitive ferroelectrics Sn_2_P_2_S_6_ with full-visible-spectrum absorption. Sci. China Mater..

[6] https://guangfu.bjx.com.cn/news/20240614/1382976.shtml.

[7] Tu Y., Wu J., Xu G., Yang X. (2021). Perovskite solar cells for space applications: Progress and challenges. Adv. Mater..

[8] Ansari M.I.H., Qurashi A., Nazeeruddin M.K. (2018). Frontiers, opportunities, and challenges in perovskite solar cells: A critical review. J. Photochem. Photobiol. C.

[9] Elumalai N.K., Mahmud M.A., Wang D. (2016). Perovskite solar cells: Progress and advancements. Energies.

[10] Lin L., Yang Z., Jiang E., Wang Z., Yan J. (2019). ZnO-modified anode for high-performance SnO_2_-based planar perovskite solar cells. ACS Appl. Energy Mater..

[11] Hu X., Wang H., Wang M., Zang Z. (2020). Interfacial defects passivation using fullerene-polymer mixing layer for planar-structure perovskite solar cells with negligible hysteresis. Sol. Energy.

[12] Park S.Y., Zhu K. (2022). Advances in SnO_2_ for efficient and stable n-i-p perovskite solar cells. Adv. Mater..

[13] Fujiwara H. (2007). Spectroscopic Ellipsometry: Principles and Applications.

[14] Tompkins H., Irene E.A. (2005). Handbook of Ellipsometry.

[15] Aspnes D.E. (2014). Spectroscopic ellipsometry-past, present, and future. Thin Solid Films.

[16] Anaya M., Lozano G., Calvo M.E., Miguez H. (2017). ABX_3_ perovskites for tandem solar cells. Joule.

[17] Mao X., Sun L., Wu T., Chu T., Deng W., Han K. (2018). First-principles screening of all-inorganic lead-free ABX_3_ perovskites. J. Phys. Chem. C.

[18] Yoo J.J., Shin S.S., Seo J. (2022). Toward efficient perovskite solar cells: Progress, strategies, and perspectives. ACS Energy Lett..

[19] Tan S., Huang T., Yavuz I., Wang R. (2022). Stability-limiting heterointerfaces of perovskite photovoltaics. Nature.

[20] Subedi B., Song Z., Chen C., Li C. (2021). Optical and electronic losses arising from physically mixed interfacial layers in perovskite solar cells. ACS Appl. Mater. Interfaces.

[21] Li H., Cui C., Xu X., Bian S., Ngaojampa C., Ruankham P., Jaroenjittchai A.P. (2020). A review of characterization of perovskite film in solar cells by spectroscopic ellipsometry. Sol. Energy.

[22] Soldera M., Taretto K. (2018). Combining thickness reduction and light trapping for potential efficiency improvements in perovskite solar cells. Phys. Status Solidi A.

[23] Dorywalski K., Maciejewski I., Krzyżyński T. (2016). Spectroscopic ellipsometry technique as a materials characterization tool for mechatronic systems-The case of composition and doping concentration monitoring in SBN crystals. Mechatronics.

[24] Hilfiker J.N., Hong N., Schoeche S. (2022). Mueller matrix spectroscopic ellipsometry. Adv. Opt. Technol..

[25] Dorywalski K., Schmidt-Gründ R., Grundmann M. (2020). Hybrid GA-gradient method for thin films ellipsometric data evaluation. J. Comput. Sci..

[26] Dorywalski K., Lupicka O., Grundmann M., Sturm C. (2022). Combination of a global-search method with model selection criteria for the ellipsometric data evaluation of DLC coatings. Adv. Opt. Technol..

[27] Sun Z., Lian J., Gao S., Wang X., Wang Y., Yu X. (2014). Complex refractive index and thickness characterization based on ant colony algorithm and comprehensive evaluation function. J. Comput. Theor. Nanosc..

[28] Mohrmann J., Tiwald T.E., Hale J.S., Hilfiker J.N., Martin A.C. (2020). Application of a B-spline model dielectric function to infrared spectroscopic ellipsometry data analysis. J. Vac. Sci. Technol. B.

[29] Papadopoulou A., Saha R.A., Pintor-Monroy M.I., Song W., Lieberman I., Solano E. (2024). In situ annealing effect on thermally co-evaporated CsPbI_2_Br thin films studied via spectroscopic ellipsometry. ACS Appl. Mater. Interfaces.

[30] Wee A.T.S., Yin X., Tang C. (2022). Introduction to Spectroscopic Ellipsometry of Thin Film Materials: Instrumentation, Data Analysis, and Applications.

[31] Gu H., Zhu S., Song B., Fang M., Guo Z., Chen X., Zhang C., Jiang H., Liu S. (2020). An analytical method to determine the complex refractive index of an ultra-thin film by ellipsometry. Appl. Surf. Sci..

[32] Nestler P., Helm C.A. (2017). Determination of refractive index and layer thickness of nm-thin films via ellipsometry. Opt. Express.

[33] Li Z., Cui C., Zhou X., Bian S. (2022). Characterization of amorphous carbon films from 5 nm to 200 nm on single-side polished a-plane sapphire substrates by spectroscopic ellipsometry. Front. Phys..

[34] Scaramuzza D., Scaramuzza N., Ciuchi F., Versace C., Strangi G., Bartolino R. (2009). Ellipsometry investigation of the effects of annealing temperature on the optical properties of indium tin oxide thin films studied by Drude-Lorentz model. Appl. Surf. Sci..

[35] Yang G., Tao H., Qin P., Fang G. (2016). Recent progress in electron transport layers for efficient perovskite solar cells. J. Mater. Chem. A.

[36] Pham H.M., Naqvi S.D.H., Tran H., Tran H.V., Delda J., Hong S. (2023). Effects of the electrical properties of SnO_2_ and C_60_ on the carrier transport characteristics of pin-structured semitransparent perovskite solar cells. Nanomaterials.

[37] Zhang J., Fu J., Chen Q., Ma H., Jiang Z., Zhang Z., Zhou Y., Song B. (2022). 3, 5-Difluorophenylboronic acid-modified SnO_2_ as ETLs for perovskite solar cells: PCE>22.3%, T82>3000 h. Chem. Eng. J..

[38] Hossain I.M., Hudry D., Mathies F., Abzieher T., Moghadamzadeh S. (2018). Scalable processing of low-temperature TiO_2_ nanoparticles for high-efficiency perovskite solar cells. ACS Appl. Energy Mater..

[39] Zhao P., Lin Z., Wang J., Yue M., Su J., Zhang J., Chang J., Hao Y. (2019). Numerical simulation of planar heterojunction perovskite solar cells based on SnO_2_ electron transport layer. ACS Appl. Energy Mater..

[40] Yeom E.J., Shin S.S., Yang W.S. (2017). Controllable synthesis of single crystalline Sn-based oxides and their application in perovskite solar cells. J. Mater. Chem. A.

[41] Dong Q., Shi Y., Zhang C., Wu Y., Wang L. (2017). Energetically favored formation of SnO_2_ nanocrystals as electron transfer layer in perovskite solar cells with high efficiency exceeding 19%. Nano Energy.

[42] Dong Q., Xue Y., Wang S., Wang L., Chen F., Zhang S., Chi R., Zhao L., Shi Y. (2017). Rational design of SnO_2_-based electron transport layer in mesoscopic perovskite solar cells: More kinetically favorable than traditional double-layer architecture. Sci. China Mater..

[43] Batzill M., Diebold U. (2005). The surface and materials science of tin oxide. Prog. Surf. Sci..

[44] Kuang Y., Zardetto V., Gils R.V., Karwal S., Koushik D., Verheijen M.A. (2018). Low-temperature plasma-assisted atomic-layer-deposited SnO_2_ as an electron transport layer in planar perovskite solar cells. ACS Appl. Mater. Interfaces.

[45] Jiang Q., Zhang L., Wang H., Yang X., Meng J., Liu H., Yin Z., Wu J., Zhang X., You J. (2016). Enhanced electron extraction using SnO_2_ for high-efficiency planar-structure HC (NH_2_)_2_PbI_3_-based perovskite solar cells. Nat. Energy.

[46] Yan W., Liu L., Li W., Wu Z., Zang Y., Wang Y., Liu K., Chen M., Zhong Z. (2021). Toward high efficiency for long-term stable Cesium doped hybrid perovskite solar cells via effective light management strategy. J. Power Sources.

[47] Kam M., Zhang Q., Zhang D., Fan Z. (2019). Room-temperature sputtered SnO_2_ as robust electron transport layer for air-stable and efficient perovskite solar cells on rigid and flexible substrates. Sci. Rep..

[48] Choi J., Song S., Hörantner M.T., Snaith H.J., Taiho P. (2016). Well-defined nanostructured, single-crystalline TiO_2_ electron transport layer for efficient planar perovskite solar cells. ACS Nano.

[49] Qiu L., Liu Z., Ono L.K., Jiang Y., Son D.Y., Hawash Z., He S., Qi Y. (2019). Scalable fabrication of stable high efficiency perovskite solar cells and modules utilizing room temperature sputtered SnO_2_ electron transport layer. Adv. Funct. Mater..

[50] Dong Q., Shi Y., Wang K., Li Y., Wang S., Zhang H. (2015). Insight into perovskite solar cells based on SnO_2_ compact electron-selective layer. J. Phys. Chem. C.

[51] Rus S.F., Ward T.Z., Herklotz A. (2016). Strain-induced optical band gap variation of SnO_2_ films. Thin Solid Films.

[52] Shanker G., Prathap P., Srivatsa K.M.K., Singh P. (2019). Effect of balanced and unbalanced magnetron sputtering processes on the properties of SnO_2_ thin films. Curr. Appl. Phys..

[53] Gong J., Wang X., Fan X., Dai R., Wang Z., Zhang Z., Ding Z. (2019). Temperature dependent optical properties of SnO_2_ film study by ellipsometry. Opt. Mater. Express.

[54] Uprety P., Lambright K.J., Grice C.R., Junda M.M., Giolando D.M., Podraza N.J. (2017). Morphological and optical properties of low temperature processed SnO_2_: F. Phys. Status Solidi B.

[55] Emam-Ismail M., Gharieb A.A., Moustafa S.H., Mahasen M.M., Shaaban E.R., El-Hagary M. (2021). Enhancement of multifunctional optoelectronic and spintronic applications of nanostructured Cr-doped SnO_2_ thin films by conducting microstructural, optical, and magnetic measurements. J. Phys. Chem. Solids.

[56] Sago Y., Fujiwara H. (2012). Mapping characterization of SnO_2_: F transparent conductive oxide layers by ellipsometry technique. J. Appl. Phys..

[57] Huang Y., Li S., Wu C., Wang S., Wang C., Ma R. (2020). Introduction of LiCl into SnO_2_ electron transport layer for efficient planar perovskite solar cells. Chem. Phys. Lett..

[58] Jiang Q., Zhang X., You J. (2018). SnO_2_: A wonderful electron transport layer for perovskite solar cells. Small..

[59] Luo T., Ye G., Chen X., Wu H., Zhang W., Chang H. (2022). F-doping-enhanced carrier transport in the SnO_2_/perovskite interface for high-performance perovskite solar cells. ACS Appl. Mater. Interfaces.

[60] Song S., Kang G., Pyeon L., Lim C., Lee G.Y., Park T., Choi J. (2017). Systematically optimized bilayered electron transport layer for highly efficient planar perovskite solar cells (η = 21.1%). ACS Energy Lett..

[61] Alias M.S., Dursun I., Saidaminov M.I., Diallo E.M., Mishra P., Ng T.K., Bakr O.M., Ooi B.S. (2016). Optical constants of CH_3_NH_3_PbBr_3_ perovskite thin films measured by spectroscopic ellipsometry. Opt. Express.

[62] Çetinörgü E., Goldsmith S., Rosenberg Y. (2007). Influence of annealing on the physical properties of filtered vacuum arc deposited tin oxide thin films. J. Non-Cryst. Solids.

[63] Carvalho D.H.Q., Schiavon M.A., Raposo M.T. (2012). Synthesis and characterization of SnO_2_ thin films prepared by dip-coating method. Phys. Procedia.

[64] Howari H., Tomsah I.B.I. (2017). Structural, optical and ellipsometric characteristics of PVD synthesized SnO_2_ thin films on Pt coated silicon wafers. Optik.

[65] Muhire E., Yang J., Hou X., Gao M. (2019). Dependence of electrical and optical properties of sol-gel-derived SnO_2_ thin films on Sb-substitution. Mater. Sci..

[66] Yahi A.H., Bouzidi A., Miloua R., Nakrela A., Khadraoui M., Tabet-Derraz H., Desfeux R., Ferri A., Blach J.F. (2019). The relationship between processing and structural, optical, electrical properties of spray pyrolysed SnO_2_ thin films prepared for different deposition times. Optik.

[67] Mohamed S.H. (2012). SnO_2_ dendrites-nanowires for optoelectronic and gas sensing applications. J. Alloys Compd..

[68] Jellison G.E., Hermann R.P., Specht E.D., Boatner T., Ward Z., Herklotz A. (2022). Generalized ellipsometry measurements of crystalline thin film and bulk tin oxide. Phys. Status Solidi A.

[69] Ganchev M., Katerski A., Stankova S., Eensalu J.S., Terziyska P., Gergova R., Popkirov G., Vitanov P. (2019). Spin-coating of SnO_2_ thin films. J. Phys. Conf. Ser..

[70] Manzoor S., Häusele J., Bush K.A., Palmstrom A.F. (2018). Optical modeling of wide-bandgap perovskite and perovskite/silicon tandem solar cells using complex refractive indices for arbitrary-bandgap perovskite absorbers. Opt. Express.

[71] Moradi S., Sedghi H. (2021). Spectroscopic ellipsometry investigation on optical properties of Fe: SnO_2_ thin films: Effects of iron concentration. Surf. Rev. Lett..

[72] Afzaal M., Yates H.M., Al-Ahmed A., Ul-Hamid A., Salhi B., Ali M. (2020). Understanding nanomechanical and surface ellipsometry of optical F-doped SnO_2_ thin films by in-line APCVD. Appl. Phys. A.

[73] So H.S., Park J., Jung D.H., Ko K.H., Lee H. (2015). Optical properties of amorphous and crystalline Sb-doped SnO_2_ thin films studied with spectroscopic ellipsometry: Optical gap energy and effective mass. J. Appl. Phys..

[74] Protesescu L., Yakunin S., Bodnarchuk M.I., Krieg F., Caputo R., Hendon C.H. (2015). Nanocrystals of cesium lead halide perovskites (CsPbX_3_, X = Cl, Br, and I): Novel optoelectronic materials showing bright emission with wide color gamut. Nano Lett..

[75] https://commons.wikimedia.org/wiki.

[76] Green M.A., Ho-Baillie A., Snaith H.J. (2014). The emergence of perovskite solar cells. Nat. Photonics.

[77] Dong Q., Li J., Shi Y., Chen M., Ono L.K., Zhou K., Zhang C., Qi Y., Zhou Y., Padture N.P. (2019). Improved SnO_2_ electron transport layers solution-deposited at near room temperature for rigid or flexible perovskite solar cells with high efficiencies. Adv. Energy Mater..

[78] Zhao J., Wang P., Liu Z., Wei L., Yang Z., Chen H., Fang X., Liu X., Mai Y. (2015). Controlled reaction for improved CH_3_NH_3_PbI_3_ transition in perovskite solar cells. Dalton Trans..

[79] Jamal M.S., Bashar M.S., Hasan A.K.M., Almutairi Z.A., Alharbi H.F., Alharthi N.H. (2018). Fabrication techniques and morphological analysis of perovskite absorber layer for high-efficiency perovskite solar cell: A review. Renew. Sustain. Energy Rev..

[80] Im J.H., Kim H.S., Park N.G. (2014). Morphology-photovoltaic property correlation in perovskite solar cells: One-step versus two-step deposition of CH_3_NH_3_PbI_3_. APL Mater..

[81] Rhaleb H.E., Benamar E., Rami M., Poger J.P., Hakam A., Ennaoui A. (2002). Spectroscopic ellipsometry studies of index profile of indium tin oxide films prepared by spray pyrolysis. Appl. Surf. Sci..

[82] Fujiwara H., Fujimoto S., Tamakoshi M., Kato M., Kadowaki H., Miyadera T., Tampo H., Chikamatsu H.S. (2017). Determination and interpretation of the optical constants for solar cell materials. Appl. Surf. Sci..

[83] Shirayama M., Kadowaki H., Miyadera T., Sugita T., Tamakoshi M., Kato M., Fujiseki T., Murata D., Hara S., Murakami T.N. (2016). Optical transitions in hybrid perovskite solar cells: Ellipsometry, density functional theory, and quantum efficiency analyses for CH_3_NH_3_PbI_3_. Phys. Rev. Appl..

[84] Ndione P.F., Li Z., Zhu K. (2016). Effects of alloying on the optical properties of organic-inorganic lead halide perovskite thin films. J. Mater. Chem. C.

[85] El-Naggar A.M., Osman M.M., Alanazi A.Q., Mohamed M.B., Ebdah M.A., Aldhafiri A.M., Heiba Z.K., Albrithen H.A. (2022). Spectroscopic ellipsometry and solar cell performance of Cs-doped MA_0.05_FA_0.95_Pb(I_0.98_Br_0.02_)_3_ triple cation perovskite thin films for solar cell applications. Appl. Phys. A.

[86] Zhang W., Fei T., Cheng T., Zheng C., Dong Y., Yang J., Liu L. (2021). Doping and temperature-dependent UV-Vis optical constants of cubic SrTiO_3_: A combined spectroscopic ellipsometry and first-principles study. Opt. Mater. Express.

[87] Kundu S., Kelly T.L. (2020). In situ studies of the degradation mechanisms of perovskite solar cells. EcoMat.

[88] Jiang Y., Soufiani A.M., Gentle A., Huang F., Ho-Baillie A., Green M. (2016). Temperature dependent optical properties of CH_3_NH_3_PbI_3_ perovskite by spectroscopic ellipsometry. Appl. Phys. Lett..

[89] Leguy A.M.A., Hu Y., Campoy-Quiles M., Alonso M.I., Weber O.J. (2015). Reversible hydration of CH_3_NH_3_PbI_3_ in films, single crystals, and solar cells. Chem. Mater..

[90] Raja W., Allen T.G., Said A.A., Alharbi O., Aydin E., Bastiani M.D., Wolf S.D. (2022). Temperature-dependent optical modeling of perovskite solar cells. J. Phys. Chem. C.

[91] Mannino G., Deretzis I., Smecca E., Giannazzo F., Valastro S. (2021). CsPbBr_3_, MAPbBr_3_, and FAPbBr_3_ bromide perovskite single crystals: Interband critical points under dry N_2_ and optical degradation under humid Air. J. Phys. Chem. C.

[92] Zhao M., Shi Y., Dai J., Lian J. (2018). Ellipsometric study of the complex optical constants of a CsPbBr_3_ perovskite thin film. J. Mater. Chem. C.

[93] Bailey C.G., Piana G.M., Lagoudakis P.G. (2019). High-energy optical transitions and optical constants of CH_3_NH_3_PbI_3_ measured by spectroscopic ellipsometry and spectrophotometry. J. Phys. Chem. C.

[94] Wang X., Gong J., Shan X., Zhang M., Xu Z., Dai R., Wang Z., Wang S., Fang X., Zhang Z. (2018). In situ monitoring of thermal degradation of CH_3_NH_3_PbI_3_ films by spectroscopic ellipsometry. J. Phys. Chem. C.

[95] Yan W., Guo Y., Beri D., Dottermusch S., Chen H., Richards B.S. (2020). Experimental determination of complex optical constants of air-stable inorganic CsPbI_3_ perovskite thin films. Phys. Status Solidi RRL.

[96] Wang S., Zhao K., Shao Y., Xu L., Huang Y., Li W. (2020). Evolutions of optical constants, interband electron transitions, and bandgap of Sn-doped CH_3_NH_3_PbI_3_ perovskite films. Appl. Phys. Lett..

[97] Chen C., Wu D., Yuan M., Yu C., Zhang J., Li C., Duan Y. (2021). Spectroscopic ellipsometry study of CsPbBr_3_ perovskite thin films prepared by vacuum evaporation. J. Phys. D Appl. Phys..

[98] Tejada A., Peters S., Al-Ashouri A., Turren-Cruz S.H., Abate A. (2022). Hybrid perovskite degradation from an optical perspective: A spectroscopic ellipsometry study from the deep ultraviolet to the middle infrared. Adv. Opt. Mater..

[99] Shil S.K., Wang F., Egbo K.O., Wang Y., Kwok C.K.G., Tsang S.W., Ho J.C., Yu K.M. (2023). Chemical vapor deposition growth and photodetector performance of lead-free all-inorganic crystalline Cs_3_Sb_2_X_9_ (X = I, Br) perovskite thin films. J. Mater. Chem. C.

[100] Yadav C., Kumar M., Lodhi K., Kumar S. (2023). Methyl ammonium iodide via novel PECVD process for the growth of 2-step vacuum based perovskite (MAPbI_3_) thin films. Mater. Today Commun..

[101] Villa-Bracamonte M.F., Montes-Bojorquez J.R., Ayon A.A. (2024). Optical properties study of a perovskite solar cell film stack by spectroscopic ellipsometry and spectrophotometry. Results Opt..

[102] Khan M.T., Haris M.P.U., Alhouri B., Kazim S., Ahmad S. (2024). Optical constants manipulation of formamidinium lead iodide perovskites: Ellipsometric and spectroscopic twigging. Energy Adv..

[103] Wang L., Nughays R., Rossi T.C., Oppermann M., Ogieglo W., Bian T., Shih C., Guo T., Pinnau I., Yin J. (2024). Disentangling thermal from electronic contributions in the spectral response of photoexcited perovskite materials. J. Am. Chem. Soc..

[104] Xu L., Chen X., Jin J., Liu W., Dong B., Bai X., Song H., Peiss P. (2019). Inverted perovskite solar cells employing doped NiO hole transport layers: A review. Nano Energy.

[105] Kong M., Garriga M., Reparaz J.S., Alonso M.I. (2022). Advanced optical characterization of PEDOT: PSS by combining spectroscopic ellipsometry and Raman scattering. ACS Omega.

[106] Tumen-Ulzii G., Qin C., Matsushima T., Leyden M.R., Balijipalli U., Klotz D., Adachi C. (2020). Understanding the degradation of Spiro-OMeTAD-based perovskite solar cells at high temperature. Sol. RRL.

[107] Woods-Robinson R., Fioretti A.N., Haschke J., Boccard M., Persson K.A., Ballif C. Linking simulation and synthesis of nickel oxide hole-selective contacts for silicon heterojunction solar cells. Proceedings of the 2020 47th IEEE Photovoltaic Specialists Conference (PVSC).

[108] Kim Y.C., Yang T.Y., Jeon N.J., Im J., Jang S., Shin T.J. (2017). Engineering interface structures between lead halide perovskite and copper phthalocyanine for efficient and stable perovskite solar cells. Energy Environ. Sci..

[109] Lu H.L., Scarel G., Alia M., Fanciulli M., Ding S., Zhang D. (2008). Spectroscopic ellipsometry study of thin NiO films grown on Si (100) by atomic layer deposition. Appl. Phys. Lett..

[110] Park J.H., Seo J., Park S., Shin S.S., Kim Y.C., Jeon N.J., Shin H.W. (2015). Efficient CH_3_NH_3_PbI_3_ perovskite solar cells employing nanostructured p-type NiO electrode formed by a pulsed laser deposition. Adv. Mater..

[111] Wang K., Shen P., Li M., Chen S., Lin M., Chen P., Guo T. (2014). Low-temperature sputtered nickel oxide compact thin film as effective electron blocking layer for mesoscopic NiO/CH_3_NH_3_PbI_3_ perovskite heterojunction solar cells. ACS Appl. Mater. Interfaces.

[112] Pettersson L.A.A., Carlsson F., Inganäs O., Arwin H. (1998). Spectroscopic ellipsometry studies of the optical properties of doped poly (3, 4-ethylenedioxythiophene): An anisotropic metal. Thin Solid Films.

[113] Eerden M., Jaysankar M., Hadipour A. (2017). Optical analysis of planar multicrystalline perovskite solar cells. Adva. Adv. Opt. Mater..

[114] Hasan M., Lyon K., Trombley L., Smith C., Zakhidov A. (2019). Thickness measurement of multilayer film stack in perovskite solar cell using spectroscopic ellipsometry. AIP Adv..

[115] Zhumagali S., Isikgor F.H., Maity P., Yin J., Ugur E., Bastiani M.D. (2021). Linked nickel oxide/perovskite interface passivation for high-performance textured monolithic tandem solar cells. Adv. Energy Mater..

[116] Shim J.W., Fuentes-Hernandez C., Dindar A., Zhou Y., Khan T.M., Kippelen B. (2013). Polymer solar cells with NiO hole-collecting interlayers processed by atomic layer deposition. Org. Electron..

[117] Jafarzadeh F., Castriotta L.A., Calabrò E., Spinelli P. (2024). Optimized NiOx deposition for industrial compatible perovskite solar modules on 15 × 15 cm^2^: A green route towards stable methylammonium-free devices. Res. Square.

[118] Mateos-Anzaldo D., Nedev R., Perez-Landeros O., Curiel-Alvarez M., Castillo-Saenz J. (2023). High-performance broadband photodetectors based on sputtered NiOx/n-Si heterojunction diodes. Opt. Mater..

[119] Steirer K.X., Richards R.E., Sigdel A.K., Garcia A., Ndione P.F., Mammond S. (2015). Nickel oxide interlayer films from nickel formate-ethylenediamine precursor: Influence of annealing on thin film properties and photovoltaic device performance. J. Mater. Chem. A.

[120] Filipič M., Löper P., Niesen B., Wolf S.D., Krč J., Ballif C., Topič M. (2015). CH_3_NH_3_PbI_3_ perovskite/silicon tandem solar cells: Characterization based optical simulations. Opt. Express.

[121] Girtan M., Mallet R., Socol M., Stanculescu A. (2020). On the physical properties PEDOT: PSS thin films. Mater. Today Commun..

[122] Sittinger V., Schulze P.S.C., Messmer C., Pflug A., Goldschmidt J.C. (2022). Complex refractive indices of Spiro-TTB and C_60_ for optical analysis of perovskite silicon tandem solar cells. Opt. Express.

[123] Zhang C., Ji C., Park Y., Guo L.J. (2021). Thin-metal-film-based transparent conductors: Material preparation, optical design, and device applications. Adv. Opt. Mater..

[124] Hilfiker J.N., Singh N., Tiwald T., Convey D., Smith S.M., Baker J.H., Tompkins H.G. (2008). Survey of methods to characterize thin absorbing films with spectroscopic ellipsometry. Thin Solid Films.

[125] Gong J., Dai R., Wang Z., Zhang Z. (2015). Thickness dispersion of surface plasmon of Ag nano-thin films: Determination by ellipsometry iterated with transmittance method. Sci. Rep..

[126] Sundari S.T., Chandra S., Tyagi A.K. (2013). Temperature dependent optical properties of silver from spectroscopic ellipsometry and density functional theory calculations. J. Appl. Phys..

[127] Singh R.K., Kumar R., Jain N., Dash S.R., Singh J., Srivastava A. (2019). Investigation of optical and dielectric properties of CsPbI_3_ inorganic lead iodide perovskite thin film. J. Taiwan Inst. Chem. Eng..

[128] Ma L., Xu X., Cui C., Lou S., Qin Y., Scott P., Zeng W. (2024). Enhancing ellipsometry analysis with whale optimisation: A novel approach for precise material characterisation. Proc. CIRP.

[129] Ma L., Xu X., Cui C., Gao M., Li T., Lou S., Scott P., Jiang X., Zeng W. (2025). A whale optimization algorithm-based data fitting method to determine the parameters of films measured by spectroscopic ellipsometry. Photonics.

[130] Patel S.J., Jariwala A., Panchal C.J., Kheraj V. (2020). Determination of thickness and optical parameters of thin films from reflectivity spectra using teaching-learning based optimization algorithm. J. Nanoelectron. Phys..

[131] Arunachalam A., Berriel S.N., Feit C., Kumar U., Seal S., Basu K., Banerjee P. (2022). Machine learning approach to thickness prediction from in situ spectroscopic ellipsometry data for atomic layer deposition processes. J. Vac. Sci. Technol. A.

